# Molecular mechanism of gut microbiota regulation in skeletal muscle metabolic remodeling and meat quality trait formation in livestock and poultry: a review

**DOI:** 10.3389/fmicb.2026.1896270

**Published:** 2026-08-18

**Authors:** Lin Han, Xuan Huang, Qi Chen, Rui Zhi, Shanshan Li, Qiong Peng, Honglin Li, Zhongyou Peng, Min Qi, Runqi Fu, Yanlong Qiao

**Affiliations:** 1 Department of Animal Husbandry and Fisheries, Guizhou Vocational College of Agriculture, Guiyang, China; 2 Faculty of Animal Science and Technology, Yunnan Agricultural University, Kunming, China; 3 Key Laboratory of Animal Nutrition and Feed Science of Yunnan Province, Yunnan Agricultural University, Kunming, China; 4 Yunnan Animal Husbandry Station, Kunming, Yunnan, China

**Keywords:** gut-muscle axis, intramuscular fat, meat quality traits, metabolic products, muscle fiber type, skeletal muscle metabolism

## Abstract

Meat quality is a major determinant of consumer acceptance and economic value, as meat serves as a primary source of high quality protein and essential micronutrients. The formation of meat is a highly complex biological process orchestrated by genetic background, nutritional regulation, and microbial modulation. Recent advances in sequencing technologies and microbiome research have elucidated the regulatory role of the gut microbiota in skeletal muscle metabolism and meat quality via the gut-muscle axis. This review comprehensively synthesizes variations in the gut microbiota of livestock and poultry, highlighting their associations with key meat quality traits such as color, water-holding capacity, tenderness, flavor, and fat deposition. We critically examine how the gut microbiota influences skeletal muscle metabolism and meat formation through the modulation of lipid, glucose, and protein metabolism, alongside muscle fiber characteristics. Furthermore, we review major microbial-derived mediators and signaling pathways integral to gut-muscle crosstalk—specifically short-chain fatty acids, bile acids, amino acids, and vitamins—and discuss how microbial homeostasis or dysbiosis impacts muscle mass and function via inflammatory, oxidative, and hormonal mechanisms. Collectively, these insights clarify potential mechanisms underlying the gut microbiota and muscle axis in animals. Consequently, this review provides a robust theoretical framework for future mechanistic studies and offers practical guidance for developing microbiota-targeted strategies to enhance muscle health and meat quality in livestock and poultry production systems.

## 1 Introduction

Meat, as a primary source of high-quality protein in the human diet, provides complete proteins containing essential amino acids as well as a wide range of key micronutrients, thereby occupying a central position in human nutrition and health. As production efficiency increases, consumer preference continues shifting toward quality rather than mere quantity, establishing meat quality as the primary driver of market value. The development of meat relies on complex biological processes encompassing coordinated biochemical reactions alongside the molecular regulation of adipocyte and myocyte proliferation (Han et al., 2025). Early investigations largely prioritized genetic dissection and dietary optimization to understand the regulatory mechanisms dictating meat traits (Han et al., 2025; Yang et al., 2025). The rapid evolution of high throughput sequencing and bioinformatics has transitioned microbiome research from descriptive profiling to functional characterization. This shift allows for a systematic evaluation of how microbial communities regulate host physiology and influence meat quality at the molecular level (Wen et al., 2023). The intestine functions not only as a central organ for digestion and immunity but also harbors a highly complex ecosystem of bacteria, archaea, fungi, and protozoa. These symbiotic microorganisms critically support nutrient metabolism, energy balance, and immune homeostasis (Chew et al., 2023). Gut microbes exert extensive control over host health by maintaining intestinal stability and functional integrity (Chew et al., 2023). Within livestock production, intestinal microbiota facilitate the fermentation of fibrous feed into bioavailable nutrients and animal protein, providing a critical biological foundation for efficient meat yield (Han et al., 2025). While several reviews address the relationship between intestinal microbes and meat quality, their scope remains restricted. For instance, Wen et al. (2024) focused exclusively on poultry, and Liu et al. (2026) limited their analysis to pigs. Furthermore, He et al. (2025) examined the interaction between intestinal microbes and muscle across species but failed to systematically link microbial metabolites with specific meat quality traits. Crucially, none of these prior works integrated the mechanistic roles of microbial metabolites such as short chain fatty acids (SCFAs), bile acids, branched chain amino acids, and vitamins with meat formation across livestock and poultry, highlighting the necessity of this comprehensive synthesis.

As research in this field has deepened, the regulatory influence of the gut microbiota on the muscular system has become increasingly apparent, leading to the emergence of the concept of the gut-muscle axis (He et al., 2025). This conceptual framework posits that gut microbial communities modulate skeletal muscle mass, myofiber type composition, functional capacity, and overall physiological health through a diverse array of microbial metabolites and signaling mediators (Chew et al., 2023; Yan et al., 2025). Accumulating evidence has demonstrated that microbial homeostasis or dysbiosis significantly influences muscle quality, myofiber type composition, and functional outcomes, including muscle strength and endurance (Lahiri et al., 2019; Yan et al., 2016, 2025; Xiong et al., 2024; Chen et al., 2024; Han et al., 2025). More recent studies have further suggested that the gut microbiome plays pivotal roles in key meat quality traits, including intramuscular fat (IMF) deposition and myofiber-type switching (Krause et al., 2020; Song et al., 2023, 2025; Han et al., 2025). Through the gut-muscle axis, microbial homeostasis or disruption can broadly reshape host energy metabolism, secondary metabolite production, endocrine signaling, and immune homeostasis, thereby remodeling skeletal muscle structure and function (Giron et al., 2022; Wang et al., 2023; Yan et al., 2025). Dysregulation of this axis has also been closely associated with pathological conditions such as sarcopenia (Mancin et al., 2023) and muscle atrophy (Lahiri et al., 2019). Among microbially derived bioactive molecules, short chain fatty acids (SCFAs) have been shown to maintain muscle satellite cell homeostasis, mitigate muscle atrophy, and regulate neuromuscular junction integrity (Lahiri et al., 2019; Frampton et al., 2020). In parallel, other microbial metabolites, including bile acids (Chen et al., 2025), branched chain amino acids (BCAAs, Yang et al., 2025), and vitamins (Gao et al., 2022), act as signaling ligands or metabolic substrates that engage specific receptors and downstream pathways, thereby orchestrating skeletal muscle physiology and metabolic regulation across multiple functional layers. It should be noted that ruminants possess a specialized rumen harboring dense anaerobic communities that produce volatile fatty acids as the predominant energy source. This represents a quantitatively distinct route of microbial energy supply compared with monogastric livestock and poultry. Nevertheless, the core metabolite driven regulatory logic, whereby microbial fermentation products engage host receptor networks to modulate skeletal muscle metabolism, is fundamentally conserved across species, even as the relative contributions of individual pathways vary. Although existing studies have outlined the interaction framework between gut microbes and skeletal muscle, systematic dissection of the regulatory network governing meat formation and translation of these insights into practical production strategies remain major challenges.

Given the complex interplay among gut microbiota, skeletal muscle function, and meat quality, this review aimed to systematically summarize the structure and function of the gut-muscle axis in livestock and poultry. We synthesized current evidence on gut microbial diversity and its regulatory roles in meat quality trait development, and we delineated the molecular mechanisms by which gut microbiota modulated skeletal muscle metabolism through lipid, carbohydrate, and protein metabolic pathways, as well as through regulation of muscle fiber type composition. In addition, we summarized the modes of action of key microbial mediators, including SCFAs, bile acids, BCAAs, and vitamins. On this basis, we identified critical knowledge gaps in gut muscle axis research and outlined future priorities for mechanistic dissection and translational implementation.

## 2 Variation in the gut microbiota across livestock and poultry and its relationship with meat quality

Meat quality is a multidimensional trait encompassing muscle mass and sensory attributes such as pH, color, drip loss, and marbling (Han et al., 2025; Xiong et al., 2024; Table 1). These traits are shaped by genetic background, diet, and management practices and are closely associated with muscle structure, chemical composition, and gut microbiota profiles (Joo et al., 2013). With advances in microbiome research, the link between gut microbial variation and meat quality has attracted increasing attention. The gut represents a complex and dynamic ecosystem composed of bacteria, fungi, protozoa, and archaea (Xiong et al., 2024). Marked differences in microbial community structure have been observed among livestock and poultry breeds, and such variation has been considered a key biological basis for diversity in meat quality traits (Han et al., 2025; Wu et al., 2024; Yang et al., 2025; Song et al., 2025). Early studies reported distinct fiber degrading microbial profiles between animals with divergent body conformation or adiposity phenotypes, suggesting that the gut microbiota indirectly influences meat quality by modulating dietary fiber utilization and host energy metabolism (Han et al., 2025). Subsequent studies showed higher abundances of *Lactobacillus* in the jejunum of Meishan pigs, the ileum and colon of Ningxiang pigs, and the colon of Xiangcun Black and Bama Miniature pigs compared with Duroc × Landrace × Large White (DLY) pigs (Wu et al., 2024; Yang et al., 2025; Song et al., 2025). In addition, lactic acid bacteria such as *HT002, Ligilactobacillus*, and *Limosilactobacillus* in the ileal mucosa of Ningxiang pigs, as well as fiber-degrading taxa including *Spirochaeta* and *Bacillus* in the colon of Jinwu pigs, were more abundant than in DLY pigs (Yang et al., 2025). Enrichment of these microbial taxa was associated with improved intestinal barrier function and immune homeostasis and with enhanced SCFAs production and amino acid metabolism, thereby promoting lipid oxidation and flavor precursor accumulation, including free amino acids (glutamate, glycine, and alanine), branched-chain amino acid-derived aldehydes (3-methylbutanal and 2-methylbutanal), and lipid oxidation-derived aldehydes (hexanal and nonanal), and ultimately improving meat flavor and tenderness (Song et al., 2025). In poultry, gut microbial variation is similarly correlated with meat quality traits. Specifically, *Microbacterium* and *Sphingomonas* exhibited greater abundance in chickens with higher body weights, whereas *Slackia* predominated in individuals with lower body weights, indicating that specific microbial taxa are linked to muscle phenotypes associated with growth (Zhang et al., 2022). Collectively, these findings demonstrate that interspecies and interbreed differences in gut microbial diversity and dominant taxa parallel the variation in meat quality phenotypes, providing robust evidence for the linkage between the gut and meat quality.

**Table 1:** Gut microbiota associated with meat-quality traits in livestock.

Livestock breed	Microbial taxon	Meat quality-related trait	References
Pigs	*Lactobacillus* spp., *Akkermansia, Clostridium_sensu_stricto_1, Stenotrophomonas, Pyramidobacter piscolens, Sphaerochaeta globosa, Lactobacillus reuteri*, HT002*, Ligilactobacillus, Limosilactobacillus, Prevotella* spp., *Clostridium* spp., *Escherichia coli*., *P. copri*	Improve lipid deposition potential and IMF; Improved muscle quality and enhanced meat flavor attributes	Tang et al., 2020; Wu et al., 2021, 2024; Xie et al., 2021; Yin et al., 2023; Yang et al., 2025; Song et al., 2025; He et al., 2025; Li et al., 2026
Cattle	*Faecalibacterium, Romboutsia, Akkermansia, Paeniclostridium, Roseburia, Prevotella, Angustibacter, Faecalibacterium, Roseburia inulinivorans, Eubacterium rectale, Clostridium puranum, Cnuella, Gaetbulibacter, Moheibacter, Lacibacter, Moheibacter*	Increased beef redness (a^*^), Higher IMF content, improved oxidative muscle metabolism and meat tenderness; linked with favorable lipid deposition patterning and quality-associated phenotypes.	Whon et al., 2021; Fan et al., 2019; Zheng et al., 2022; Xiong et al., 2024; Wang H. et al., 2021; Zhang et al., 2025
Sheep	*Phocaeicola, Christensenellaceae_R-7_group, Ruminococcus* spp.	Muscle lightness (L^*^) and postmortem color stability.	Wang H. et al., 2021
Chicken	*Lactobacillus plantarum, Akkermansia muciniphila, Faecalibacterium prausnitzii*	Reduced muscle glycolytic capacity, lower postmortem pH decline, Increased slow oxidative muscle fibers and improved tenderness and WHC.	Yan et al., 2025; Cai et al., 2024; Wang et al., 2025; Yang et al., 2021
Duck	*Lactobacillus* spp., *Bacillus subtilis*	Improved muscle energy metabolism and meat color stability.	Chen et al., 2025

Although the gut-muscle axis plays a role in all livestock and poultry species, pigs, ruminants, and poultry exhibit significant differences in gut structure and microbial composition, resulting in regulatory mechanisms that are both common and species-specific. Ruminants possess a multi-chambered forestomach; the rumen harbors a complex anaerobic community comprising bacteria, protozoa, fungi, and archaea, which produce SCFAs through fermentation as the host's primary energy source (Han et al., 2025). Pigs, as monogastric animals, undergo microbial fermentation primarily in the hindgut (cecum and colon). The quantitative contribution of SCFAs to the body's energy homeostasis is relatively limited, whereas the bile acid-FXR/TGR5 and BCAAs signaling pathways play a more prominent role in gut-muscle communication (Tardiolo et al., 2025). Poultry, on the other hand, possess a unique avian digestive tract, with paired caeca serving as the primary site of fermentation. The gut microbiota is dominated by members of the genus Lactobacillus and the phylum Firmicutes, and the rapid passage of chyme results in a unique metabolite profile (Burrows et al., 2025; Aruwa et al., 2021). Therefore, although microbial metabolites such as SCFAs, bile acids, and amino acid derivatives regulate skeletal muscle lipid and protein metabolism in all species, there are significant interspecies differences in the quantitative contributions of each pathway. Specifically, SCFAs-driven energy supply predominates in ruminants, hindgut SCFAs and bile acid signaling is more critical in pigs, and cecal fermentation shapes muscle metabolism in poultry in a species-specific manner.

### 2.1 Regulation of meat quality traits by the gut microbiota in livestock and poultry

The composition and dynamic remodeling of the gut microbiota in livestock and poultry can regulate key meat quality traits through multiple direct and indirect pathways. In ruminants, increased relative abundances of bacterial genera such as *Romboutsia, Akkermansia*, and *Paeniclostridium* in the bovine intestine were significantly correlated with higher beef redness (a^*^) values (Han et al., 2025). The reduced abundances of *Phocaeicola* and the *Christensenellaceae_R-7_group* were associated with decreased muscle lightness (L^*^) at both 45 min and 24 h postmortem in Goat (Wang K. et al., 2021). Beyond natural microbial variation, targeted interventions including fecal microbiota transplantation (FMT) and probiotic supplementation have been shown to reshape gut microbial communities and improve meat quality. Transplantation of Beijing broiler microbiota into Arbor chickens altered intestinal *Lachnoclostridium* abundance and was accompanied by increased body weight, reduced drip loss, and enhanced abdominal fat deposition (Lei et al., 2022). Dietary probiotic supplementation in chickens, pigs, cattle and sheep consistently improved meat color, reduced drip loss (Chen et al., 2018; Wang et al., 2022), and modulated pH and shear force (Soumeh et al., 2021). In addition, the gut microbiota significantly influenced meat chemical composition (Liu et al., 2022, 2023) and the profiles of flavor related compounds (Soumeh et al., 2021; Liu et al., 2023). Collectively, these findings indicated that associations between the microbiota and meat quality are broadly conserved across species, underscoring the translational potential of microbiome based strategies for improving meat quality.

#### 2.1.1 Regulation of intramuscular fat deposition by the gut microbiota

**Figure 1:**
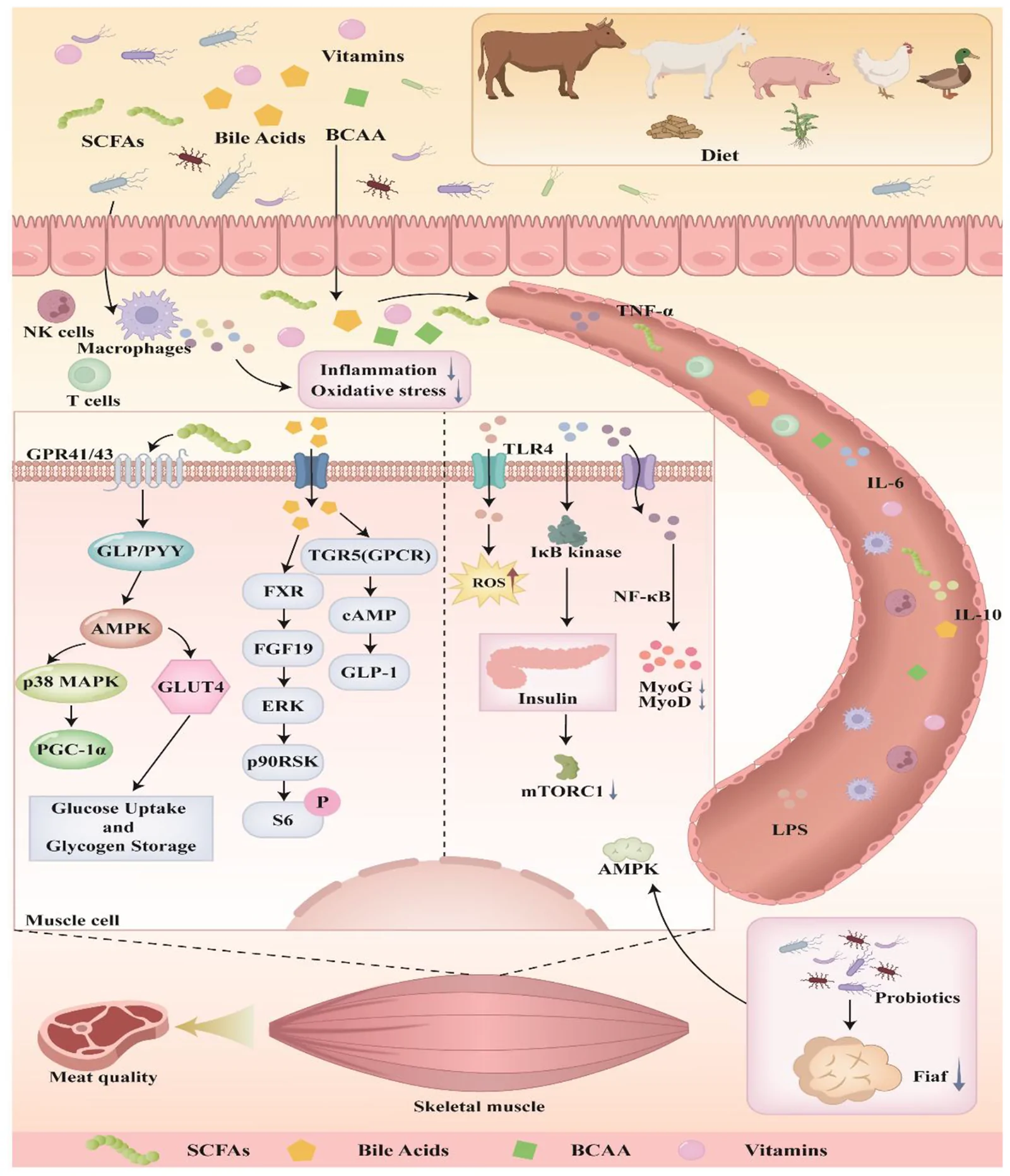
The gut microbiota of livestock and poultry co-regulates skeletal muscle metabolic homeostasis via microbially derived metabolites and immuno-inflammatory cues, thereby shaping meat quality phenotypes. The microbiota produces short-chain fatty acids (SCFAs), bile acids, branched-chain amino acids (BCAAs), and vitamins that cross the intestinal epithelium into the portal and/or systemic circulation to act on peripheral tissues. SCFAs signal through GPR41/43 to engage GLP/PYY-associated pathways and activate the AMPK–p38 and MAPK–PGC-1α program, enhancing mitochondrial oxidative metabolism, while promoting glucose uptake and glycogen storage through GLUT4. Bile acids signal via FXR to induce FGF19 and activate ERK, leading to phosphorylation of p90RSK and ribosomal protein S6, which supports protein synthesis and muscle growth; they also activate the GPCR TGR5 to elevate cAMP and facilitate GLP-1-linked metabolic regulation. Under barrier disruption or dysbiosis, lipopolysaccharide (LPS) translocates into the circulation and triggers a TLR4-IκB kinase–NF-κB inflammatory cascade with reactive oxygen species accumulation, accompanied by shifts in cytokine outputs (e.g., TNF-α, IL-6, IL-10), which perturb insulin signaling and metabolic hubs such as mTORC1 and AMPK and modulate myogenic regulators (e.g., MyoD, MyoG) and protein turnover. Immune cells (T cells, macrophages, and NK cells) contribute to intestinal inflammatory and oxidative milieus and can influence muscle metabolism through systemic inflammatory signaling. Microbiome-targeted interventions (e.g., probiotics) can remodel microbial communities and affect Fiaf/ANGPTL4-related pathways, thereby altering lipid partitioning and energy substrate use. Collectively, these metabolic and immune pathways converge on muscle metabolic phenotypes and structural function and align with meat quality traits, including color, WHC, tenderness, flavor, and fat deposition.

Fat deposition is a complex physiological process that is strongly influenced by the structure and metabolic capacity of the gut microbiota (Figure 1 and Tables 2, 3). Obesity-related phenotypes have been closely associated with specific microbial signatures, and microbial dysbiosis has been linked to lipid metabolic disorders and aberrant fat accumulation (Zhao, 2013). Because microbial communities associated with IMF and subcutaneous fat do not fully overlap, gut microbes are thought to regulate these depots through partially distinct lipogenic pathways (Krause et al., 2020). Among adipose tissues, IMF plays a decisive role in meat tenderness, juiciness, and flavor, making it a key economic trait. Beyond age, sex, and nutritional inputs, host genetic background and the gut microbiome jointly governed intramuscular fat deposition, which exhibited stage specific sensitivity and pronounced genetic nutritional interactions during meat quality development (Zheng et al., 2022; Song et al., 2025). Moreover, breed dependent differences in muscle fiber composition and metabolic phenotype modulate lipid oxidation and deposition capacity, thereby contributing to variation in intramuscular fat content and associated meat quality traits. Accumulating evidence in pigs, cattle, and other livestock has demonstrated close associations between specific gut microbes and IMF content (Lahiri et al., 2019; Tang et al., 2020; Han et al., 2025; Song et al., 2025). Segmental analyses of the porcine intestine identified jejunal and cecal microbiota as strongly correlated with backfat thickness and IMF, with *Prevotellaceae_UCG-001, Akkermansia*, and *Clostridium_sensu_stricto_1* showing positive associations, whereas Stenotrophomonas was negatively associated (Tang et al., 2020). Comparative analyses across pig breeds further identified numerous IMF-associated OTUs, primarily from Bacteroidetes, Spirochaetes, Prevotellales, and Clostridiales, which are enriched in amino acid metabolism and polysaccharide degradation functions (Fang et al., 2017). High *Prevotella* abundance was shown to promote adipogenesis by upregulating lipogenic genes, accumulating branched-chain and aromatic amino acids, and suppressing lipolysis-related pathways (He et al., 2023; Liang et al., 2025). Similarly, elevated Firmicutes/Bacteroidetes ratios and increased *Romboutsia* abundance were associated with higher IMF levels (Wu et al., 2021). Integrated with evidence from poultry models, these findings indicated that microbiota mediated regulation of intramuscular fat predominantly operates through lipid synthesis and transport pathways in concert with muscle metabolic state, thereby providing a coherent framework for reconciling divergent microbiota intramuscular fat associations reported across studies (Song et al., 2025).

**Table 2:** Summary of recent studies on the effects of probiotic supplementation on meat quality.

Livestock breed	Probiotic strains	Intervention dosage	Animal age/body weight	Experimental duration	Meat quality-related trait	References
Pig	*Lactobacillus johnsonii* (BNCC135265)	Mono-colonization	28.6 kg DLY pigs	14 d	Increased total fatty acids and saturated fatty acids	Ma et al., 2022
	*B. laterosporus* BL1	0.5% diet	69.7 kg DLY pigs	71 d	Improve antioxidant capacity, free amino acid profiles, and fatty acid composition	Weng et al., 2025
	*Prevotella copri* (DSM 18205)	1.0 × 10^10^ CFU/kg feed	61 kg fattening pigs	45 d	Improving meat color and reduction in drip loss	Liang et al., 2025
Sheep	*L. casei* HM-09 and *L. plantarum* HM-10	1.5 × 10^9^ CFU/g	3-month-old Sunit sheep; 19.03 ± 3.67 kg	90 d	Promoted oxidative myofiber conversion; decreased shear force and cooking loss	Wang et al., 2022
	*Lactiplantibacillus plantarum* N-1	1 × 10^9^ CFU/g/kg BW	4.72 ± 0.61 kg	60 d	Increased gumminess	Zhou et al., 2023
	*Clostridium butyricum*	2.5 × 108 CFU/g, 5 g/day/lamb	88 ± 5 d; 27.43 ± 1.94 kg	90 d	Increased growth performance, muscle mass, fiber diameter and CSA; decreased shear force	Dou et al., 2023

**Table 3:** Summary of recent studies on the effects of probiotic supplementation on meat quality.

Livestock breed	Probiotic strains	Intervention dosage	Animal age/body weight	Experimental duration	Meat quality-related trait	References
Chicken	*Lactiplantibacillus plantarum* PA01 fermented feed	3% fermented feed	1-day-old male Arbor Acre broilers	42 d	Improved meat quality and lipid metabolism; reduced shear force and increased IMF	Wang et al., 2026
	*L. plantarum, L. salivarius, L. ingluviei*, or mixed suspension	10^8^ CFU/ml	22-day-old Arbor Acres chickens	21 d	Increased fiber diameter and fiber density of pectoralis muscle	Cai et al., 2024
	*Brevibacillus laterosporus* S62-9	10^6^ CFU/g	1-day-old male Arbor Acres broilers	42 d	Decreased pH, pressing loss, cooking loss, and shear force	Liu et al., 2023
Duck	Bacillus, Lactobacillus, Pediococcus, yeast	0.4–0.5 g/kg	1-day-old Muscovy ducks	60 d	Improved flavor, acceptability and WHC	Mohammed et al., 2026
Cattle	6 mixed strains	2 g/100 kg	Newborn/40 kg	7 months	Improved loin muscle area and marbling	Gandra et al., 2022

Fecal microbiota transplantation experiments provided causal support for these associations. Transplantation of microbiota from fat-type Jinhua pigs into mice increased lipid accumulation, triglyceride levels, and lipoprotein lipase activity while reducing *ANGPTL4* expression in muscle, compared with microbiota from lean-type Landrace pigs (Wu et al., 2021). Similarly, transferring Large White pig microbiota into DLY pigs enhanced IMF deposition in the longissimus dorsi muscle, with *Bacteroides uniformis, Pyramidobacter piscolens*, and *Sphaerochaeta globosa* identified as key *adipogenic* species that upregulated *Cd36, Dgat2*, and *FASN* (Xie et al., 2021). Ningxiang pig microbiota further promoted muscular fatty acid deposition by modulating carnitine metabolism (Yin et al., 2023). In ruminants, gut microbes also played a critical role in fat deposition. Increased abundances of *Roseburia, Prevotella*, and *Angustibacter* were associated with higher intramuscular fat content in beef cattle, whereas the energy regulating genus *Akkermansia* was enriched in low intramuscular fat Brahman calves, and the butyrate producing genus *Faecalibacterium* showed a positive correlation with intramuscular fat deposition in Angus cattle (Fan et al., 2019; Zheng et al., 2022; Xiong et al., 2024). In contrast, *Tenericutes* and *Saccharibacteria* were negatively associated with IMF content (Krause et al., 2020). Moreover, species such as *Roseburia inulinivorans, Eubacterium rectale*, and *Clostridium puranum* correlated with muscle metabolic genes including *MSTN, ATP2A1*, and *MYLPF*, thereby contributing to favorable beef quality traits (Zheng et al., 2022). Collectively, these findings establish the gut microbiota as a key regulator of IMF deposition and fat distribution in livestock and poultry, underscoring its central role in meat quality formation.

#### 2.1.2 Regulatory mechanisms of gut microbiota and muscle mass

Muscle mass relies on the quantity and dimensions of muscle fibers alongside components lacking contractile ability, such as connective tissue, functioning as a primary determinant of physical strength and functional capacity (Marullo and O'Halloran, 2023; Figure 1). Variation in intestinal microbial composition among individuals dictates nutrient absorption and metabolic efficiency to direct muscle growth and maintenance (He et al., 2025). Recent investigations reveal that pigs experience compromised skeletal muscle development compared with control groups free from specific pathogens. This impairment is evidenced by reduced myofiber diameter and a diminished proportion of type I fibers (Li C. et al., 2025). In contrast, dietary supplementation with probiotics such as *Lactobacillus reuteri* increased muscle mass and improved meat flavor in pigs (He et al., 2025). Consistent observations in rodent models showed that germ free (GF) or antibiotic treated mice displayed reduced muscle mass and contractile strength, phenotypes that were reversible following microbiota transplantation or natural recolonization, concomitant with the restoration of myosin heavy chain (*MyHC*) isoform expression and metabolic homeostasis (Lahiri et al., 2019; Nay et al., 2019; Li C. et al., 2025). These observations indicate that, beyond genetic and nutritional factors, the gut microbiota acts as a critical regulator of muscle mass in livestock and poultry. Gut dysbiosis is frequently associated with impaired intestinal barrier integrity and increased translocation of lipopolysaccharide into the circulation, which activates inflammatory signaling pathways such as *NF-*κ*B* and elevates proinflammatory cytokines including TNF-α and IL-6. This inflammatory cascade induces the expression of muscle atrophy related ubiquitin ligases such as *Atrogin-1* and *MuRF1*, suppresses protein synthesis and mitochondrial biogenesis via pathways including mTORC1, AMPK and PGC-1α, and ultimately compromises skeletal muscle mass and contractile function (Wang H. et al., 2021; Wang et al., 2023, 2025; Yang et al., 2025). Lipopolysaccharide-driven inflammation and oxidative stress further exacerbate muscle weakness, fatigue, and atrophy (Fang et al., 2021; Yu et al., 2024). In contrast, butyrate producing bacteria, exemplified by *Faecalibacterium prausnitzii*, generate butyrate and other anti-inflammatory metabolites, promote IL-10 signaling, and suppress *NF-*κ*B* activation. These bacteria are widely recognized as key indicators of gut health and systemic anti-inflammatory status, and their enrichment correlates with enhanced intestinal barrier integrity and reduced systemic inflammation, thereby providing a protective environment for skeletal muscle maintenance (Lenoir et al., 2020). Randomized controlled trials further showed that probiotic supplementation, such as *Lactobacillus plantarum TWK10*, improved muscle strength and endurance in elderly individuals and animal models, in parallel with increased abundance of SCFAs producing microbes, enhanced muscle glycogen storage, and attenuation of chronic inflammation (Lee et al., 2021). In livestock, dietary probiotic supplementation reshaped gut microbiota composition and modulated amino acid and fatty acid profiles in muscle by regulating genes associated with muscle growth and flavor, thereby influencing meat quality attributes (Cai et al., 2021; Tables 2, 3). Collectively, these findings suggested that dietary strategies or microbiota-targeted interventions have strong potential to optimize muscle mass, immune balance, and production performance in livestock.

## 3 Gut microbiota-mediated mechanisms of skeletal muscle metabolism

### 3.1 Gut microbiota regulation of skeletal muscle lipid metabolism

Accumulating evidence indicated that the gut microbiota plays a central role in systemic lipid metabolism, partly through coordinated regulation of skeletal muscle lipid utilization pathways (Xiao et al., 2024; Figure 1). Among these mechanisms, fasting-induced adipose factor (*Fiaf* ), also known as *ANGPTL4*, represents a key molecular node linking the intestinal environment to lipoprotein lipase activity. Gut microbiota suppresses *ANGPTL4* expression, thereby enhancing triglyceride-rich lipoprotein hydrolysis and increasing fatty acid availability to peripheral tissues, including skeletal muscle (Nay et al., 2019). Because *ANGPTL* family members govern tissue specific triglyceride partitioning by modulating lipoprotein lipase activity, microbiota driven regulation of *ANGPTL4* alters fatty acid flux into muscle and shapes metabolic flexibility. Integrated models further highlighted the coordinated control of lipid utilization by *ANGPTL3, ANGPTL4*, and *ANGPTL8* as a core mechanism underlying peripheral lipid metabolism (Gugliucci, 2024; Ramos et al., 2025). At the intracellular level, *ANGPTL4* associated changes interact with energy sensing pathways, particularly AMPK, to regulate fatty acid oxidation programs. These effects involve modulation of key enzymes such as carnitine palmitoyltransferase 1 and medium chain acyl-CoA dehydrogenase, thereby influencing the intensity of fatty acid catabolism in skeletal muscle (Bäckhed et al., 2007; Qi et al., 2022). GF and antibiotic-treated animal models supported a causal contribution of gut microbiota to muscle lipid metabolism, although reported changes in *Cpt1b* and *Mcad* expression or activity varied across studies, reflecting strong context dependence related to diet, developmental stage, muscle type, and microbial colonization patterns (Bäckhed et al., 2007; Lahiri et al., 2019; Qi et al., 2022). Proteomic analyses further showed that gut microbiota can suppress AMPK signaling while promoting fatty acid synthase expression, thereby enhancing lipid storage capacity in myocytes (Porstmann et al., 2008). In livestock systems, feeding regimes shape gut microbial composition and, in turn, influence skeletal muscle lipid quality. For example, specific dietary patterns in sheep improved muscle lipid oxidative stability through microbiota mediated metabolic pathways (Wen et al., 2024). Recent studies on lambs have shown that adding 1% L-arginine to the diet can reshape the colonic microbiota and differentially regulate lipid metabolism in subcutaneous adipose tissue (SAT) and the longissimus thoracis (LT) muscle through the GLP-1R/AMPK and triglyceride metabolism signaling pathways, This is manifested by a reduction in backfat thickness and saturated fatty acid content in subcutaneous adipose tissue (SAT), as well as an increase in IMF and polyunsaturated fatty acid (PUFA) deposition in the longissimus thoracis (LT) muscle, thereby improving meat tenderness (Kang et al., 2025). These results suggest that microbiota-derived SCFAs and enteric endocrine signals such as *GLP-1* may serve as key mediators linking dietary interventions, lipid redistribution in ruminant muscle, and improvements in meat quality. The fatty acid translocase *CD36*, a major sarcolemmal transporter, shows a strong positive association between its expression and muscle lipid uptake, and its role within the microbiota-muscle metabolic axis has been increasingly recognized (Bonen et al., 2004; Xie et al., 2021). In pigs, post weaning microbiota dynamics correlated closely with metabolic shifts that affected muscle lipid balance and meat related phenotypes (Li et al., 2024). Similarly, diet induced microbiome remodeling in meat geese was accompanied by AMPK activation and Akt-mTOR pathway modulation, coinciding with improved muscle metabolic traits (Zulfiqar et al., 2025). Specifically, AMPK functions as a central cellular energy sensor that, upon phosphorylation at Thr172, directly phosphorylates PGC-1α to promote mitochondrial biogenesis and oxidative fiber formation, while concurrently inactivating acetyl-CoA carboxylase to relieve CPT1 inhibition and enhance fatty acid β-oxidation, thereby improving muscle oxidative capacity and metabolic flexibility (Jäger et al., 2007; Steinberg and Hardie, 2023). In parallel, Akt-mediated activation of mTORC1 promotes ribosome biogenesis and cap-dependent translation initiation through phosphorylation of S6K1 and 4E-BP1, stimulating myofibrillar protein synthesis, while Akt-dependent phosphorylation and inactivation of FoxO transcription factors suppresses the expression of Atrogin-1 and MuRF1 ubiquitin ligases, thereby reducing proteolytic degradation. The coordinated enhancement of protein synthesis and attenuation of protein degradation collectively drive skeletal muscle hypertrophy and mass maintenance (Bodine et al., 2001). Thus, the gut microbiota-AMPK and -Akt/mTOR axes represent complementary regulatory modules, AMPK governing oxidative metabolic capacity and Akt/mTOR governing anabolic protein balance, that synergistically improve muscle growth and metabolic quality in livestock and poultry (Zulfiqar et al., 2025). Consequently, the microbiota-AMPK and microbiota-Akt/mTOR axes function as complementary regulatory modules, the former dictates oxidative metabolic capacity, whereas the latter controls anabolic protein homeostasis. These pathways act in synergy to promote muscle growth and elevate metabolic quality in livestock and poultry. Collectively, the gut microbiota orchestrates a multilevel regulatory network that governs skeletal muscle lipid metabolism by integrating ANGPTL4-dependent lipid partitioning, AMPK-centered energy sensing, GLP-1R-mediated enteroendocrine signaling, and CD36-mediated fatty acid transport. This mechanistic framework lays a critical foundation for formulating microbiota-targeted nutritional strategies to optimize muscle metabolism and improve meat quality in agricultural production.

### 3.2 Gut microbiota regulation of skeletal muscle glucose metabolism

Skeletal muscle is the largest site of glucose disposal in the body, and its glycogen stores constitute a critical metabolic reservoir (Aliani et al., 2013; Figure 1). The rate of glycogen turnover directly determines the extent of postmortem glycolysis, thereby shaping pH decline and ultimately influencing final meat quality (Aliani et al., 2013; Figure 1). Excessive glycogen accumulation accelerates post-slaughter acidification and predisposes meat to defects such as pale, soft, and exudative phenotypes. Growing evidence indicated that the gut microbiota regulates skeletal muscle glucose uptake and utilization predominantly through microbially derived metabolites, particularly SCFAs (Han et al., 2014). SCFAs stimulate the secretion of glucagon like peptide-1 and peptide YY via *GPR41* and *GPR43*, thereby activating AMPK signaling and improving systemic and muscular glucose homeostasis (Giron et al., 2022). Lactic acid bacteria, including *Lactobacillus plantarum, Lactobacillus reuteri*, and *Lactobacillus salmonensis*, enhanced skeletal muscle glucose utilization by increasing SCFAs production and upregulating key metabolic enzymes such as phosphofructokinase, pyruvate kinase, pyruvate dehydrogenase, isocitrate dehydrogenase, and succinate dehydrogenase, collectively limiting excessive glycogen deposition (Cai et al., 2024). In addition, microbiota-derived metabolites such as 3-O-sulfate isovanillic acid activated the PI3K-AKT pathway, promoted *GLUT4* expression, and increased glucose uptake and oxidative utilization in myocytes (Houghton et al., 2019). Consistent with these findings, GF models showed that microbiota depletion reduced SCFAs availability in skeletal muscle while increasing the accumulation of organic acids such as pyruvate and fumarate, accompanied by perturbations in AMPK signaling and carbohydrate metabolism pathways, supporting a direct role of gut microbes in muscle glucose homeostasis (Li Z. et al., 2025). Collectively, the gut microbiota exerts multifaceted control over skeletal muscle glucose uptake, utilization, and glycogen metabolism. Future studies should prioritize the identification of functional microbial strains and the dissection of strain specific mechanisms governing muscle glucose metabolism.

### 3.3 Gut microbiota regulation of skeletal muscle protein metabolism

Skeletal muscle mass and composition are maintained by a dynamic balance between protein synthesis and degradation, and disruption of this balance commonly leads to muscle atrophy and functional decline (Bindels et al., 2012). Accumulating evidence indicated that gut microbiota modulated skeletal muscle protein metabolism through inflammatory, metabolic, and nutrient-sensing pathways. Microbial-derived inflammatory mediators, including LPS, TNF-α, and IL-6, suppressed mTORC1 signaling in myocytes, thereby reducing protein synthesis capacity (Wang et al., 2023). Concurrently, these signals interfered with Akt activity, attenuating its repression of *FoxO* transcription factors and leading to increased expression of the ubiquitin ligases *Atrogin-1* and *MuRF1*, which activate the ubiquitin-proteasome system (Wang et al., 2023). Specifically, these muscle-specific E3 ligases polyubiquitinate key myofibrillar and contractile proteins (such as myosin heavy chain), targeting them for rapid degradation by the 26S proteasome and consequently driving muscle protein breakdown and atrophy (Bodine et al., 2001; Wang et al., 2023). Beyond inflammatory regulation, the gut microbiota influenced protein anabolism by modulating amino acid availability and transporter expression. Specific Bacteroidetes taxa were shown to upregulate BCAAs transporters in myocytes, facilitating *mTORC1* activation and enhancing protein synthesis (Lahiri et al., 2019). Metabolomic analyses revealed elevated concentrations of amino acids such as alanine and glycine in skeletal muscle of GF mice, although their microbial contribution and metabolic fate remain to be clarified (Lahiri et al., 2019; Yang et al., 2021). In broiler chickens, gut microbial composition closely correlated with muscle amino acid profiles, the *Parabacteroides* abundance showed positive associations with methionine but negative associations with glycine and tyrosine, whereas *Achromobacter* correlated positively with tyrosine and *Stenotrophomonas* negatively with serine. Gut dysbiosis further altered skeletal muscle proteomic profiles, characterized by downregulation of protein synthesis-related components such as ribosomal protein S6 kinase and upregulation of catabolic regulators including *FOXO1*, thereby accelerating muscle protein turnover (Sandri et al., 2004). Collectively, current evidence supports a regulatory role of the gut microbiota in muscle amino acid supply, protein synthesis, and degradation. However, the identification of key functional strains and the delineation of their precise molecular pathways remain critical objectives for future research.

### 3.4 Gut microbiota regulation of muscle amino acid and fatty acid metabolism

The gut microbiota can influence the accumulation and conversion of flavor precursors in meat by regulating amino acid and fatty acid metabolism in skeletal muscle. Studies have shown that gut microbes can directly metabolize diet-derived and endogenous amino acids in the intestinal lumen, thereby reshaping the host's amino acid metabolism and further affecting their deposition in skeletal muscle (Li et al., 2024). Researchers have demonstrated that *Bacteroides* and *Clostridium* species possess BCAAs transaminase and decarboxylase activities, which convert leucine, isoleucine, and valine into their corresponding α-keto acids and branched-chain aldehydes, which are key intermediates in the Strecker degradation pathway and contribute to the formation of characteristic malty and cheesy aromas during cooking (Li et al., 2024). Consistent with this, *Lactobacillus roei* from Ningxiang pigs can regulate IMF deposition through BCAAs metabolism, and the resulting changes in the amino acid profile are closely associated with alterations in the composition of muscle flavor precursors (Yang et al., 2025). Furthermore, the gut microbiome composition of broiler chickens is closely associated with the muscle amino acid profile. Specifically, the abundance of Bacteroides is positively correlated with methionine content, and methionine is an important precursor of sulfur-containing flavor compounds. Other microbial communities are associated with amino acids such as glycine, tyrosine, and serine, all of which contribute to the formation of sweet and umami flavors (Yang et al., 2021). Dietary supplementation with *Bacillus laterosporus S62-9* increased the levels of flavor-enhancing amino acids such as glutamic acid and aspartic acid in chicken meat, while simultaneously increasing the levels of volatile compounds in breast muscle (Liu et al., 2023). Similarly, the addition of *Bacillus laterosporus S62-9* to the diets of finishing pigs improved the free amino acid profile and fatty acid composition of the pectoralis maximus muscle, indicating that probiotic-mediated microbiota modulation can simultaneously expand the pool of flavor precursors derived from both amino acids and fatty acids (Weng et al., 2025). Notably, gut microbiota can also regulate fatty acid metabolism through multiple interrelated pathways, thereby promoting the formation of lipid-derived flavor precursors. Studies have shown that the addition of lactic acid bacteria can reshape the cecal microbiota and enhance meat flavor by regulating fatty acid metabolic pathways in the gut and muscle, thereby establishing a link between microbiota-mediated lipid metabolism and the production of volatile flavor compounds (Xu et al., 2024). In ruminants, rumen microbiota-host interactions can regulate intramuscular fat deposition in cattle via the α-linolenic acid-fatty acid β-oxidation/L-carnitine–MPO axis, and further shape the fatty acid composition that serves as a substrate for the production of volatile flavor compounds (Zhang et al., 2025). Studies have found that sheep rumen microbiota can activate PPARγ signaling to regulate the fatty acid profile in muscle, directly influencing the formation of lamb flavor (Pan et al., 2025). Furthermore, microbiota-derived SCFAs can regulate *de novo* fatty acid synthesis and the ratio of saturated to polyunsaturated fatty acids by activating AMPK and PPARγ pathways in skeletal muscle, thereby contributing to the formation of flavor precursors (Yin et al., 2023; Ruan et al., 2024). Multi-omic analysis of different cattle breeds further revealed that superior meat quality is associated with higher levels of flavor-enhancing amino acids, polyunsaturated fatty acids, and a characteristic gut microbiome (Han et al., 2025). Furthermore, probiotic supplementation in lambs increased the content of volatile flavor compounds in muscle, accompanied by enhanced antioxidant capacity and altered muscle fiber characteristics, suggesting that microbiota-mediated redox regulation may maintain the stability of flavor precursors by limiting oxidative degradation (Liu et al., 2022). In summary, the gut microbiota collectively drives the formation and maintenance of meat flavor precursors by synergistically regulating skeletal muscle amino acid catabolism, fatty acid transport and oxidation, intramuscular fat deposition, and redox homeostasis.

### 3.5 Gut microbiota regulation of muscle fiber-type plasticity

Skeletal muscle is a highly heterogeneous and plastic tissue composed of fiber types with distinct metabolic and contractile properties. Based on myosin heavy chain (*MyHC*) isoforms, muscle fibers are classified as type I (slow oxidative), type IIa (fast oxidative), type IIx (intermediate), and type IIb (fast glycolytic) (Yan et al., 2025). In livestock and poultry, a higher proportion of oxidative fibers (type I and IIa) is generally associated with favorable meat quality traits, including darker color, improved tenderness, and reduced drip loss, whereas enrichment of glycolytic fibers, particularly type IIb, is linked to paler color and lower water-holding capacity (WHC, Choe and Kim, 2014; Weng et al., 2022; Gao et al., 2022).

In chickens, the breast muscle consists almost exclusively of type IIb fibers with limited breed variation, while the thigh muscle contains predominantly type IIb fibers with a minor proportion of type I fibers (Verdiglione and Cassandro, 2013; Kui et al., 2022). Muscles enriched in glycolytic fibers undergo rapid postmortem glycolysis and pH decline due to accelerated lactate accumulation, leading to protein denaturation and compromised WHC and tenderness (Weng et al., 2022). Increasing evidence indicated that the gut microbiota represents an important exogenous regulator of muscle fiber composition, influencing myofiber differentiation through microbial metabolites and signaling pathways. Compared with commercial pig breeds, indigenous breeds with superior meat quality, such as Taoyuan Black Pig and Xiangcun Black Pig, exhibited both higher proportions of oxidative fibers and increased colonic abundance of *Lactobacillus*, suggesting a potential role of this genus in promoting slow-twitch fiber formation (Song et al., 2023, 2025). In *MSTN*^−^*/*^−^ pigs, transplantation of donor microbiota increased muscle fiber cross-sectional area and the proportion of fast glycolytic fibers in recipients (Luo et al., 2023). Conversely, transfer of colonic microbiota from Rongchang pigs to GF mice increased slow-twitch fiber proportion, reduced fiber diameter, and decreased type IIb fiber abundance (Yan et al., 2016). These effects were accompanied by reciprocal regulation of *Myh7* and Myh4, encoding slow- and fast-twitch *MyHC* isoforms, respectively (Lahiri et al., 2019). Mechanistically, *Akkermansia muciniphila* was shown to promote *Myh7* expression and suppress fast-twitch fiber formation through *METTL3* dependent m^6^A methylation, providing direct molecular evidence for a gut microbiota muscle fiber regulatory axis (Yan et al., 2025). Consistently, GF pigs exhibited impaired muscle development, characterized by reduced myofibril diameter and decreased type I fiber proportion, along with marked alterations in muscle proteomic and metabolomic profiles (Li Z. et al., 2025). Independent of fiber type composition, shear force negatively correlated with muscle fiber density, indicating that, under a fixed fiber type framework, moderate increases in fiber number without excessive hypertrophy favor meat tenderness (Han et al., 2025). Overall, the gut microbiota emerges as a key determinant of muscle fiber type distribution and morphological characteristics, providing novel biological targets for optimizing meat quality through microbiota-directed nutritional and management strategies.

## 4 Key microbial metabolites mediating gut-muscle crosstalk

Although the studies summarized above provide important insights into how the gut microbiota influences meat quality traits, much of the current evidence remains correlative, and definitive causal relationships are not yet fully established. Elucidating the mechanisms by which the gut microbiota regulates skeletal muscle physiology and metabolism has therefore become a central focus of recent research. Overall, gut microorganisms primarily exert their effects through the production of bioactive metabolites, including SCFAs, bile acids, BCAAs, and vitamins, which enter the circulation and function as metabolic substrates or signaling molecules in peripheral tissues (Han et al., 2025), thereby exerting either beneficial or adverse effects on skeletal muscle. In contrast, relatively few studies have addressed how muscle-derived factors reciprocally shape gut microbial structure and function. The following sections focus on these key mediators and discuss their mechanistic roles within the gut-muscle axis.

### 4.1 Short chain fatty acid mediated regulation of the gut microbiota skeletal muscle axis

SCFAs are key functional metabolites produced by anaerobic gut microorganisms. While they mainly comprise acetate, propionate, and butyrate derived from the fermentation of indigestible dietary fibers, other important members include straight-chain SCFAs (e.g., valerate and caproate) and branched-chain SCFAs (BCFAs; e.g., isobutyrate and isovalerate) that are generated primarily through protein catabolism (Frampton et al., 2020). Because mammals lack endogenous enzymatic systems for degrading complex carbohydrates (Fu et al., 2025), hosts rely heavily on symbiotic gut microbes that supply diverse carbohydrate-active enzymes to cooperatively deconstruct biomass and generate SCFAs, a process particularly prominent in ruminants (Fu et al., 2025; Han et al., 2025). After luminal production, most SCFAs are absorbed by the intestinal epithelium and transported to the liver via the portal vein for further metabolism (Morrison and Preston, 2016), whereas a fraction enters the systemic circulation and acts on peripheral tissues, including skeletal muscle (Xiong et al., 2024; Hou et al., 2024). Notably, SCFAs produced in the hindgut can partially bypass hepatic metabolism and directly enter the circulation through the iliac vein (van der Beek et al., 2017). In addition, the three major SCFAs can be converted in the liver into other bioactive metabolites (den Besten et al., 2013); for example, microbially derived acetate serves both as a substrate for hepatic lipogenesis and as a circulating signaling molecule with regulatory functions (Xiong et al., 2024). Accordingly, SCFAs are regarded as critical mediators linking intestinal metabolic status with peripheral organs, particularly skeletal muscle (Hou et al., 2024; Chew et al., 2023; Frampton et al., 2020).

In livestock and poultry, gut derived SCFAs play important roles in fat deposition and meat quality formation, although their effects show marked species and breed specificity. *Gayal* exhibited higher intestinal SCFAs levels than yellow cattle and showed a greater capacity for unsaturated fatty acid biosynthesis (Han et al., 2025). Under identical feeding conditions, yaks produced higher total ruminal SCFAs than yellow cattle (Zhou et al., 2018), likely due to higher abundances of rumen microbial genes involved in fiber degradation, which enhanced volatile fatty acid supply and provided substrates for tissue lipid metabolism (Louis and Flint, 2017). In pigs, gut microbiota derived SCFAs regulated skeletal muscle lipid metabolism by upregulating peroxisome proliferator activated receptor alpha expression, promoting fatty acid β-oxidation, and reducing intramuscular lipid accumulation, thereby improving muscle energy utilization efficiency (Jiao et al., 2020). Importantly, elevated SCFAs levels do not necessarily correspond to increased IMF content. Observations in *Gayal* relative to yellow cattle, as well as in Shaziling pigs, suggested that SCFAs effects on lipid metabolism and fat distribution are constrained by host genetics, energy allocation strategies, and microbial community structure (Han et al., 2025; Ma et al., 2022). Conversely, other studies reported positive associations between IMF content and SCFAs levels (Xiong et al., 2024), indicating strong context dependence. Intervention studies provided stronger causal evidence. Antibiotic treatment and fecal microbiota transplantation in mice indicated that alterations in gut SCFAs levels were consistently associated with increased muscle and peri-muscular fat deposition in castrated cattle (Whon et al., 2021). In pigs, silage feeding enhanced intestinal SCFAs production and coincided with improvements in meat color, marbling, WHC, and fatty acid composition, accompanied by microbial functional enrichment in amino acid metabolism and SCFAs producing pathways (Niu et al., 2023). Collectively, these findings suggested that SCFAs contribute to meat quality phenotypes by modulating energy substrate supply and tissue metabolic partitioning, although their precise effects require stratified validation under defined genetic and production conditions.

SCFAs also participate in regulating skeletal muscle homeostasis and disease-related phenotypes. GF mice exhibited skeletal muscle atrophy accompanied by downregulation of genes involved in muscle growth and mitochondrial function, whereas supplementation with an SCFAs mixture partially alleviated these phenotypes, highlighting the importance of SCFAs in maintaining muscle structure and metabolic capacity (Lahiri et al., 2019). In sarcopenia, gut dysbiosis frequently co-occurs with abnormal metabolic signaling and reduced abundance of SCFAs producing bacteria, suggesting that SCFAs related pathways represent a key node in disrupted gut-muscle communication (Mancin et al., 2023). Beyond total SCFAs levels, individual SCFAs exert distinct effects. Early studies showed that increased dietary acetate supply correlated with elevated muscle glycogen content in pigs, supporting muscle growth through enhanced energy reserves (Imoto and Namioka, 1983). In GF mice, acetate supplementation effectively alleviated microbiota depletion-induced muscle growth retardation. Specifically, it upregulated key myogenic marker genes (such as *Myod1* and *Myog*) and significantly enhanced succinate dehydrogenase activity, thereby driving oxidative metabolic reprogramming and restoring overall skeletal muscle development (Yang et al., 2024). Butyrate promoted myocyte differentiation and protein synthesis through histone deacetylase inhibition and receptor mediated signaling (Han et al., 2025). In aged female mice, butyrate supplementation alleviated hindlimb muscle atrophy and improved locomotor performance (Walsh et al., 2015; Li C. et al., 2025). Moreover, butyrate treatment correlated with enhanced mitochondrial function, improved ATP supply, and reduced oxidative stress and apoptosis markers (Al-Harbi et al., 2018; Yoon et al., 2021), potentially supporting neuromuscular junction integrity and contractile function.

At the molecular level, expression of the SCFAs receptors *GPR41* and *GPR43* in skeletal muscle provides a mechanistic basis for tissue-specific SCFAs signaling (Han et al., 2014; Maruta and Yamashita, 2020). SCFAs function both as signaling ligands activating muscle energy sensing networks and as metabolic substrates contributing to oxidative energy production. Previous studies demonstrated that SCFAs induce AMPK phosphorylation in myotubes and skeletal muscle cells, subsequently activating p38 MAPK and PGC-1α signaling to enhance mitochondrial biogenesis and oxidative capacity (Jäger et al., 2007; Liu et al., 2024). PGC-1α is preferentially expressed in slow-twitch oxidative fibers, and its upregulation is closely associated with oxidative fiber formation and mitochondrial proliferation (Guerfali et al., 2007). Consistently, SCFAs supplementation increased PGC-1α mRNA and protein levels in L6 myotubes and skeletal muscle tissue, in parallel with enhanced slow-oxidative phenotypes (Pan et al., 2015; Maruta and Yamashita, 2020). AMPK activation also upregulates *GLUT4* and regulates transcription of energy metabolism genes by promoting histone deacetylase nuclear export (McGee et al., 2008; Steinberg and Hardie, 2023). Enhanced *GLUT4* mediated glucose uptake increases muscle glycogen storage, thereby influencing post-mortem glycolysis and meat quality traits (Richter and Hargreaves, 2013; Richter et al., 2025). Together, SCFAs constitute a central chemical communication axis linking gut microbiota and skeletal muscle through coordinated receptor signaling and energy metabolism networks. Their roles in regulating muscle metabolism and meat quality in livestock warrant further precise quantification using standardized interventions and integrative multi-omics approaches (Frampton et al., 2020).

### 4.2 Bile acid mediated regulation of the gut microbiota skeletal muscle axis

Bile acids are synthesized from cholesterol in hepatocytes through multiple enzymatic reactions and constitute amphiphilic molecules with both surfactant and signaling functions. They facilitate lipid emulsification and absorption while acting as signaling ligands that regulate lipid and energy metabolism and skeletal muscle development via receptor mediated pathways (Vítek and Haluzík, 2016). Among bile acid receptors, the FXR is a central nuclear receptor widely expressed in the liver and skeletal muscle and mediates transcriptional regulation (Zhou and Anakk, 2022). Activation of FXR and its downstream targets participates in skeletal muscle differentiation, proliferation, and hypertrophy (Chen et al., 2024, 2025). Gut microbiota enhanced skeletal muscle lipid metabolic pathways by modulating bile acid metabolism, promoting fatty acid oxidation and muscle development (Li et al., 2026). Recent evidence further indicated that bile acids not only regulate overall lipid absorption but also selectively influence uptake of specific fatty acids, thereby reshaping circulating lipid profiles and tissue lipid oxidation (Chan et al., 2026).

Bile acids enhance protein synthesis by activating FXR signaling and inducing fibroblast growth factor 19 expression, which subsequently stimulates the ERK pathway and promotes phosphorylation of p90 ribosomal S6 kinase and ribosomal protein S6 (Kir et al., 2011), supporting muscle growth while attenuating muscle atrophy and metabolic imbalance (Guo et al., 2021; Qiu et al., 2022). Alterations in gut microbiota derived secondary bile acids in rabbits correlated with skeletal muscle lipid absorption and fatty acid utilization efficiency, thereby influencing muscle energy metabolism (Zubiri-Gaitán et al., 2024). Beyond nuclear receptors, bile acids activate the G protein–coupled receptor TGR5 to increase cAMP production and stimulate GLP-1 secretion, improving hepatic glucose and lipid metabolism in high fat diet induced obesity models (Pathak et al., 2018). TGR5 activation also upregulated nitric oxide synthase expression and nitric oxide production, contributing to antioxidant effects (Reich et al., 2017). In antibiotic induced mouse models of muscle atrophy, enhanced FXR activity promoted skeletal muscle protein synthesis (Qiu et al., 2022), further substantiating a central role for bile acid FXR signaling in the regulation of muscle metabolic homeostasis. Gut microbiota reshape primary and secondary bile acid (such as deoxycholic acid and lithocholic acid) composition through deconjugation, dehydrogenation, and 7α-dehydroxylation, thereby modulating FXR, TGR5, and downstream pathways. These interactions influence bile transport, skeletal muscle cholesterol homeostasis signaling, lipid metabolism, and fatty acid oxidation (Ma et al., 2024), while FXR signaling can reciprocally alter microbial composition and host metabolic phenotypes (Parséus et al., 2017; Chen et al., 2024). In poultry, dietary bile acid supplementation alleviated abdominal fat oxidation disturbances induced by high fat diets in ducks, increased breast muscle yield, and upregulated the expression of key lipid metabolism genes, including *PPAR*α, *CPT1*α, *NR1H4*, and *CETP* (Chen et al., 2025). In broilers, bile acid supplementation activated FXR and its downstream IGF2 axis, synergistically enhancing lipid metabolism and breast muscle growth (Chen et al., 2024). Overall, bile acid signaling exhibits bidirectional coupling with gut microbiota at metabolic and receptor levels and plays a key regulatory role in skeletal muscle metabolism and meat quality traits.

### 4.3 Branched-chain amino acid-mediated regulation of the gut microbiota–skeletal muscle axis

BCAAs including leucine, isoleucine, and valine, serve as essential substrates for skeletal muscle protein synthesis and as metabolic nodes linking nutritional status to intracellular signaling. They participate in regulating energy allocation, lipid metabolism, and tissue growth (Neinast et al., 2019). Animal studies showed that reduced dietary BCAAs supply enhanced fatty acid oxidation and suppressed hepatic lipid synthesis, whereas under specific nutritional contexts, higher BCAAs intake correlated with increased lipid synthesis and storage, indicating strong context dependence (Bai et al., 2015; Surugihalli et al., 2023). In pigs, dietary BCAAs supplementation increased IMF content by modulating lipid metabolism related gene expression and by enhancing transport and utilization of circulating fatty acids in skeletal muscle (Yang et al., 2025). BCAAs, particularly leucine, also function as nutrient signals that activate the mTOR pathway, promoting muscle protein synthesis and influencing adipocyte differentiation and lipogenesis (Green et al., 2016; Hey et al., 2021). Consistently, supplementation with BCAAs was shown to attenuate exercise induced or stress related muscle damage and to enhance skeletal muscle protein synthesis capacity (Kato et al., 2015; Roberson et al., 2018). Notably, individual BCAAs differ in metabolic fate and signaling effects, resulting in non-identical regulation of muscle growth and meat quality traits (Yu et al., 2021). Leucine supplementation primarily promoted muscle protein synthesis via mTOR signaling (Green et al., 2016) and, under low-protein diets, increases protein and IMF deposition while improving pork WHC (Zhang et al., 2016). Isoleucine upregulated *GLUT1* and *GLUT4* in skeletal muscle and *SGLT1* and *GLUT2* in the intestine, enhancing glucose transport to support muscle and intestinal growth, whereas valine and isoleucine synergistically improved physicochemical meat quality traits, including WHC (Xu et al., 2020). Beyond dietary supply, gut microbiota possess BCAAs biosynthetic and conversion capacities. Differences in microbial structure and function influence host BCAAs availability and metabolic routing. *Prevotella sp. MSX73* and *Prevotella sp. MA2016* have been characterized as possessing robust biosynthetic capacities for BCAAs, thereby acting as direct microbial contributors to the host's luminal amino acid pool (Hai et al., 2024*)*. In pigs, gut microbiota regulated skeletal muscle lipid metabolism by modulating BCAAs oxidation and interacting with receptor signaling networks, thereby influencing the balance between fatty acid utilization and protein synthesis (Li Z. et al., 2025). Emerging evidence further suggested coupling between microbial BCAAs metabolism and bile acid signaling, potentially influencing skeletal muscle energy metabolism and lipid oxidation via TGR5 related pathways and contributing to growth and meat quality phenotypes in poultry (Zhang et al., 2025). Collectively, gut microbiota-mediated BCAAs metabolism shapes skeletal muscle metabolic states and meat quality traits through coordinated nutrient signaling and substrate flux.

### 4.4 Vitamin-mediated regulation of the gut microbiota–skeletal muscle axis

Vitamins play essential roles in gut microbiota–skeletal muscle interactions in livestock and poultry by shaping microbial structure and supporting muscle anabolic metabolism and functional maintenance. Dietary vitamin D supplementation enhanced meat oxidative stability, nutritional value, tenderness, and sensory quality, likely through promoting muscle protein deposition and strengthening enzymatic and non-enzymatic antioxidant systems (Półtorak et al., 2017; Gao et al., 2022). Vitamin E improved meat color stability and WHC by inhibiting lipid and myoglobin oxidation and maintaining membrane integrity (Descalzo and Sancho, 2008). It also modulated gut microbiota composition, reduced pro-inflammatory taxa, enhanced muscle antioxidant capacity, and supported protein synthesis (Agnoletti et al., 2022). In pigs, vitamin E supplementation improved feed intake, feed efficiency, and oxidative stress markers while reshaping colonic microbiota, including increased *Peptococcus* abundance and improved meat quality indices such as reduced drip loss and increased yellowness value (Zhu et al., 2022). Vitamin A exhibited a consistent negative association with IMF content across multiple bovine breeds, including Wagyu, Holstein, and Angus, with supplementation reducing IMF deposition (Oka et al., 1998; Gorocica-Buenfil et al., 2007). Proposed mechanisms included inhibition of adipocyte proliferation and reduced fatty acid synthesis and insulin sensitivity (Kruk et al., 2018; Campos et al., 2020). Meta-analyses supported the overall fat-reducing effect of vitamin A but also highlighted variability across studies, suggesting modulation by genetics, diet composition, and production stage (Li et al., 2023; Yu et al., 2022). Vitamin K1 has been proposed as a microbiota-associated protective metabolite that attenuates inflammation-induced muscle damage by downregulating atrophy-related proteins (Xiao et al., 2024). In addition, gut microbiota derived B vitamins are critical for energy and protein metabolism. Folate deficiency disrupted myoblast proliferation and differentiation and was associated with aberrant DNA methylation of myoblast determination protein 1 (MyoD) and Myogenin promoters and elevated homocysteine (Hwang et al., 2018; Giguére et al., 2008; Winter et al., 2022). In poultry, microbial synthesis of riboflavin, folate, biotin, and vitamin B12 supported muscle energy metabolism and development (Wang et al., 2025). Betaine, a nutrient linked to B vitamin metabolism, regulated muscle fiber-type transition through microbiota-mediated m^6^A RNA methylation, increasing *Myh7* expression and slow witch fiber proportion (Yan et al., 2025). Overall, elucidating the coupled actions of vitamins and gut microbiota will provide a more translatable framework for nutritional strategies targeting muscle metabolism and meat quality.

### 4.5 Cytokine-mediated regulation of the gut microbiota–skeletal muscle axis

Accumulating evidence indicated that the gut microbiota modulates immune homeostasis and skeletal muscle function in livestock and poultry by reshaping cytokine profiles. In pigs, circulating IL-6 levels positively correlated with *Lactobacillus* abundance and negatively with *Faecalibacterium*, suggesting coordinated variation between microbial structure and inflammatory output (Al Bander et al., 2020). Mechanistically, gut microbes continuously interact with intestinal epithelial and mucosal immune cells, dynamically balancing pro- and anti-inflammatory cytokines. SCFAs associated signaling suppressed TNF-α and IL-6 while promoting IL-10 production, thereby dampening inflammation and improving metabolic homeostasis (Cristofori et al., 2021; Fan et al., 2023). In contrast, dysbiosis often compromised intestinal barrier integrity, allowing lipopolysaccharide translocation into circulation and elevating IL-6, TNF-α, and IL-1β, which induced skeletal muscle inflammation, oxidative stress, metabolic disruption, and impaired contractile function (Pan et al., 2021; Wang H. et al., 2021). This inflammatory cascade increased reactive oxygen species, promoted proteolysis and structural remodeling, and heightened muscle atrophy risk. Certain cytokines also supported regeneration; for example, IL-4 facilitated myoblast fusion and angiogenesis (Tu and Li, 2023). Recent studies reported enrichment of immune-related pathways in skeletal muscle of GF pigs, where reduced SCFAs levels coincided with activation of TNF-α and IL-6 signaling and with decreased myofiber diameter and type I fiber proportion, reinforcing the role of immuno-inflammatory pathways in microbiota-dependent muscle structural regulation (Li Z. et al., 2025). In poultry, microbe derived ligands activated GPR signaling, suppressed IL-6 and TNF-α, reduced oxidative stress, and supported muscle energy metabolism and growth by limiting immune-driven catabolism (Wang et al., 2025). Skeletal muscle also functions as an endocrine organ, secreting myokines such as IGF-1, IL-4, IL-6, IL-7, IL-15, and FGF-2 that exert systemic effects and modulate gut cellular communication, thereby contributing to feedback regulation of the microbiome profound muscle metabolism and function (Pedersen and Febbraio, 2012). Dietary lipid sources and the n-6:n-3 polyunsaturated fatty acid ratio further modulated cytokine signaling and systemic inflammatory tone, aligning with changes in muscle metabolism and meat quality traits (Song et al., 2024). Collectively, cytokines act as central signaling nodes linking gut microbiota and skeletal muscle, with critical implications for muscle quality development and health maintenance in livestock and poultry.

## 5 Summary and perspectives

In this review, we have systematically examined the evidence linking the gut microbiota of livestock and poultry to the gut-muscle axis and meat quality formation. We have summarized reported associations between microbial variation and key meat-quality traits, including color, WHC, tenderness, flavor, and IMF, and discussed the major mechanisms through which gut microbes regulate skeletal muscle metabolism, encompassing lipid, carbohydrate, and protein metabolism as well as muscle fiber-type plasticity. Current research has identified SCFAs, bile acids, BCAAs, vitamins, and their receptor-mediated signaling pathways as central mediators of gut microbiota-muscle crosstalk. Importantly, oxidative stress should also be regarded as a core mediator in this regulatory network. Gut dysbiosis may impair intestinal barrier integrity and increase circulating endotoxin and inflammatory signals, thereby promoting reactive oxygen species accumulation and oxidative damage in skeletal muscle. Conversely, microbiota-derived metabolites and microbiota-modulated nutrients may enhance mitochondrial oxidative capacity and antioxidant defense. Such redox regulation can influence meat color through myoglobin oxidation, WHC through oxidative modification of myofibrillar proteins, and shelf stability through lipid and protein oxidation during storage.

However, a substantial proportion of existing studies still describe correlations rather than causality. Advancing from correlation to causation therefore represents a critical next phase of research. Future efforts should focus on integrating rigorously controlled genetic, dietary, and environmental designs with GF and colonization models, fecal microbiota transplantation combined with single-strain supplementation, stable isotope tracing, and multiomics-based causal inference to establish mechanistic links. In parallel, isolating, culturing, and functionally characterizing key microbial strains will be essential to define their direct roles in muscle protein turnover, mitochondrial function, redox homeostasis, and fiber-type remodeling, as well as to evaluate reproducibility and safety in production systems. Emerging epigenetic mechanisms, including m^6^A RNA methylation, miRNAs, and extracellular vesicle-associated nucleic acid cargo, also warrant further attention. Recent evidence suggests that gut microbiota-mediated metabolic signals may regulate skeletal muscle fiber-type transition through m^6^A-dependent gene expression, while miRNA networks are closely associated with muscle growth, myofiber characteristics, and meat-quality-related phenotypes. Future studies should integrate microbiome manipulation with epitranscriptomics, small RNA profiling, and functional validation to determine whether these epigenetic layers act as causal mechanisms within the gut-muscle axis.

From an application perspective, microbiota manipulation has already shown practical potential in livestock production and poultry production, but different strategies vary greatly in maturity and feasibility. Probiotics and synbiotics are currently the most applicable approaches, with studies in pigs, sheep, poultry, and ducks showing improvements in meat color, WHC, tenderness, IMF deposition, antioxidant capacity, muscle fiber characteristics, and flavor-related traits. Prebiotics, dietary fiber, fermented feed, and silage-based nutritional strategies are also industrially attractive because they can reshape endogenous microbial fermentation and metabolite production without introducing exogenous strains, although their effects are strongly dependent on basal diet composition, breed, growth stage, and farm management. By contrast, fecal microbiota transplantation and microbiota-derived extracellular vesicles remain valuable tools for causal verification and future precision intervention, but their routine use in commercial production is still constrained by donor selection, biosafety, regulatory approval, batch consistency, and standardization. Future application should move from single-trait improvement toward multi-objective optimization that integrates growth rate, feed conversion, carcass yield, backfat thickness, IMF, meat color, WHC, tenderness, flavor, animal health, and economic return.
